# Preliminary evidence that both blue and red light can induce alertness at night

**DOI:** 10.1186/1471-2202-10-105

**Published:** 2009-08-27

**Authors:** Mariana G Figueiro, Andrew Bierman, Barbara Plitnick, Mark S Rea

**Affiliations:** 1 Lighting Research Center, Rensselaer Polytechnic Institute, Troy, NY, USA

## Abstract

**Background:**

A variety of studies have demonstrated that retinal light exposure can increase alertness at night. It is now well accepted that the circadian system is maximally sensitive to short-wavelength (blue) light and is quite insensitive to long-wavelength (red) light. Retinal exposures to blue light at night have been recently shown to impact alertness, implicating participation by the circadian system. The present experiment was conducted to look at the impact of both blue and red light at two different levels on nocturnal alertness. Visually effective but moderate levels of red light are ineffective for stimulating the circadian system. If it were shown that a moderate level of red light impacts alertness, it would have had to occur via a pathway other than through the circadian system.

**Methods:**

Fourteen subjects participated in a within-subject two-night study, where each participant was exposed to four experimental lighting conditions. Each night each subject was presented a high (40 lx at the cornea) and a low (10 lx at the cornea) diffuse light exposure condition of the same spectrum (blue, λ_max _= 470 nm, or red, λ_max _= 630 nm). The presentation order of the light levels was counterbalanced across sessions for a given subject; light spectra were counterbalanced across subjects within sessions. Prior to each lighting condition, subjects remained in the dark (< 1 lx at the cornea) for 60 minutes. Electroencephalogram (EEG) measurements, electrocardiogram (ECG), psychomotor vigilance tests (PVT), self-reports of sleepiness, and saliva samples for melatonin assays were collected at the end of each dark and light periods.

**Results:**

Exposures to red and to blue light resulted in increased beta and reduced alpha power relative to preceding dark conditions. Exposures to high, but not low, levels of red and of blue light significantly increased heart rate relative to the dark condition. Performance and sleepiness ratings were not strongly affected by the lighting conditions. Only the higher level of blue light resulted in a reduction in melatonin levels relative to the other lighting conditions.

**Conclusion:**

These results support previous findings that alertness may be mediated by the circadian system, but it does not seem to be the only light-sensitive pathway that can affect alertness at night.

## Background

Alertness is a construct associated with high levels of environmental awareness. Alertness has been operationalized through many converging measurements, including subjective responses, behavior, and brain activity. Alertness is associated with self-reported high levels of wakefulness and low levels of fatigue, short response times, fast and more accurate tests of mental capacity, low power densities in the alpha frequency range (8-12 Hz) and high power densities in the beta frequency range (12-30 Hz) in electroencephalography (EEG) [1-9]. Since these outcome measures follow diurnal patterns, alertness also can be related to measures of endogenous hormones and core body temperature; alertness is expected to be low during the nighttime hours, when melatonin levels are high and core body temperature levels are low.

In response to the natural light-dark solar patterns, humans have evolved endogenous circadian (circa = around; dies = day) rhythms (e.g., core body temperature variations, melatonin synthesis, sleep-wake behavior) that repeat approximately every 24 hours (average period of 24.2 hours). The internal mechanism that organizes these daily biological processes in mammals has been localized to small paired nuclei in the hypothalamus, the suprachiasmatic nuclei (SCN). Circadian rhythms are synchronized to the 24-hour solar day by photic and non-photic cues, the light-dark cycle being the strongest. Light and dark patterns are conveyed from the retina to the SCN via the retino-hypothalamic tract (RHT).

Alertness and performance are strongly influenced by the timing of the circadian clock; therefore, the impact of light on alertness has gained recent attention. Bright white light (2500 to 10,000 lx at the cornea) has been shown to increase alertness at night but not during the day, suggesting a role for the circadian system in evoking alertness [1-9]. An early study by Badia and colleagues [1], for example, tested the effects of bright white light (5000 to 10,000 lx at the cornea) on core body temperature, alertness, and performance during the day and at night. In their study, subjects who normally would be awake during the day and asleep at night were exposed to 90-minute blocks of alternating bright and dim (50 lx at the cornea) light during daytime and nighttime hours. Core body temperature, beta power (18-21 Hz) from the EEG recordings, and performance were higher after exposure to bright light than after exposure to dim light during the nighttime hours, but not during daytime hours. These results suggest a role for the circadian system in modulating alertness, but the mechanisms associated with the alerting effects of light through the circadian system cannot be unambiguously established by simply introducing "bright" light into the experimental protocol.

It is well established now that the human circadian system is maximally sensitive to short-wavelength radiation (blue light) [10-12]. Not only does this mean that the circadian system is quite insensitive to low levels of long-wavelength, red light, it also means on a quantitative basis that the efficacy of "dim" blue light can be computed to be equivalent to that of "bright" white light for stimulating the circadian system [12]. Cajochen and colleagues showed that low levels of blue light (5 lx at the cornea of narrowband radiation peaking at 460 nm) for a duration of about 40 minutes at night increased heart rate and self-reported alertness as measured by the Karolinska Sleepiness Scale (KSS), as well as reduced melatonin levels [13]. More recently, Figueiro and colleagues demonstrated that, at night, self-reported alertness (Norris Scale) and the alpha attenuation coefficient (AAC; the ratio of the alpha power [8-12 Hz] when eyes are closed to the alpha power when eyes are open) in the EEG recordings were highly correlated and both increased monotonically with increasing levels of narrowband blue (peak at 470 nm) light (5, 10, 20 and 40 lx at the cornea) [14]. Moreover, they found these measures of alertness were highly correlated with predicted levels of light-induced nocturnal melatonin suppression for the same light stimuli. These results are consistent with the neurophysiological evidence that neural pathways from the SCN are important to sleep and to alertness, as recently elucidated by Saper and colleagues [15-17]. Together, these findings add weight to the inference that the SCN, through retinal stimulation by short-wavelength light, play an important role in nocturnal alertness in humans.

It is not completely clear, however, whether light-induced alertness can arise from other neural pathways. For example, some evidence suggests that red light, which is ineffective for stimulating the circadian system at moderate light levels, can be more stimulating than blue light [18,19]. Studies have also suggested that perception of red color prior to executing an important task impairs performance relative to the perception of green or achromatic color [19,20]. The present experiment was conducted to look at the impact that both blue and red light at two different levels might have on human subjective and objective measures of alertness, on performance and on melatonin levels during the night.

## Methods

### Procedures and apparatus

Sixteen subjects (21 to 46 years of age) were recruited to participate in the study from an electronic posting at Rensselaer Polytechnic Institute in Troy, N.Y. All subjects were screened for major health problems and except for women taking birth control pills, subjects reported not taking any pharmaceuticals or medications. Every subject completed a Munich Chronotype Questionnaire (MCTQ) prior to the study [21]; those who were late or extremely late chronotypes were excluded from the experiment. All subjects provided an informed consent approved by Rensselaer's Institute Review Board. Subjects were asked to refrain from alcohol and caffeine on the days of the experiment and were asked not to sleep after awakening for the day. Of the sixteen subjects, nine males and five females completed the entire experiment, and the results of their data are reported here.

Four experimental lighting conditions, two spectra (blue and red) at two levels (10 and 40 lx), were delivered to individual subjects from 0.6 × 0.6 × 0.6 m light boxes, each fitted with arrays of light-emitting diodes (LEDs). The arrays (ICove, Color Kinetics) were located behind the front box apertures to be outside the subject's direct view, thereby creating a uniform, non-glaring distribution of light within the box. During light exposures, subjects placed their chin on a rest mounted near the front of the box, ensuring delivery of the prescribed light exposure. The spectral emissions of the blue LEDs peaked at 470 nm with a full width at half maximum (FWHM) of 25 nm. Light from the red LEDs peaked at 630 nm with a FWHM of 25 nm. Before the experiment, each of the light boxes was calibrated using a Gigahertz illuminance photometer to provide the prescribed corneal illuminance levels when subjects were positioned in the chinrest. The spectral radiances of the red and blue conditions were measured prior to the experiment with a calibrated spectroradiometer (Photoresearch, model PR705a) and diffuse white reflectance standard (Labsphere, model SR 099) and were used to calibrate the Gigahertz illuminance readings. Two boxes provided blue light (40 μW/cm^2 ^at 40 lx and 10 μW/cm^2 ^at 10 lx) and two emitted red light (19 μW/cm^2 ^at 40 lx and 4.7 μW/cm^2 ^at 10 lx); light levels could be adjusted with an electronic dimmer to reach the prescribed light levels without significantly affecting the relative spectral distributions of the LED emissions. Measurements of pupil area completed after the experiment with a different group of subjects (n = 5) were: red at 10 lx, 34 mm^2^; red at 40 lx, 22 mm^2^; blue at 10 lx, 10 mm^2^; blue at 40 lx, 6.5 mm^2^.

**Figure 1:**
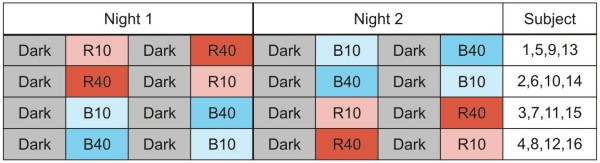
**Experimental design**. Each subject participated in the experiment over the course of two nights. On night 1, subjects saw two light levels (10 lux or 40 lux) of the same light spectra, either blue (B) or red (R) following a dark (D) condition; subjects experienced the other light spectra at both light levels on night 2. Note: Subjects 12 and 13 did not complete the experiment, so their data are not presented here.

**Figure 2:**
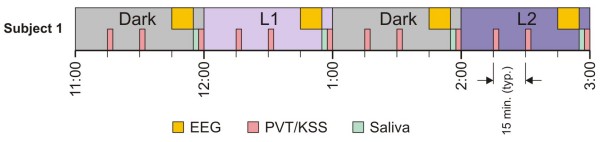
**Data collection times for the first subject on an experimental night**. During a 1-hr period, PVT and KSS data were collected twice during the first 45 minutes. In the last 15 minutes, EEG, saliva and a third PVT and KSS measures were collected. Four subjects were run each experimental night; start times for subsequent subjects were staggered by 15 minutes.

The study was conducted over the course of several weeks in April/May 2008 and in March 2009. Groups of four subjects participated in two sessions separated by at least one week. Subjects were asked to arrive at the laboratory at 22:00 to receive instructions, become familiar with the performance tests, and be fitted with scalp electrodes for EEG recordings. Because only one EEG machine was available, data collection was staggered. The first subject in a session started at 23:00, the second at 23:10, the third at 23:20, and the last at 23:30; the last subject completed the experiment at 03:45. During every session, each subject was presented a high (40 lx) and a low (10 lx) light exposure condition of the same spectrum (blue or red). The presentation order of the light levels was counterbalanced across sessions for a given subject; light spectra were counterbalanced across subjects within sessions. Every 45-minute light exposure condition was preceded by a 45-minute period of inactivity in a dark anteroom (< 1 lx of red light at the cornea). During the inactive dark periods, subjects remained quiet and were not allowed to perform any task (e.g., talk, read, or computer work) except for the prescribed data sampling specified in the experimental protocol. Each data collection period (after each dark and after each light exposure condition), which included EEG measurements, saliva collection and PVT tests, lasted about 15 minutes. In sum then, in each nighttime session there was a total of four, 45-minute light-and-dark conditions (a dark condition always preceded one of the four light conditions), and each condition was continued for 15 minutes for data collection. Figures 1 and 2 show the experimental design and the data collection activity periods, respectively.

### PVT and KSS

Self-luminous personal digital assistants (PDA; Tungsten E2 models from Palm Inc.) were used to record subjects' performance levels and sleepiness self-reports [22,23]. A battery of three psychomotor vigilance tests (PVT; Brain Checkers 2.75 from Behavioral Neuroscience Systems LLC) were used to measure performance: (a) a Simple Reaction Times (RT) test where the subject had to tap a symbol displayed on the PDA screen with a stylus as soon it appeared; (b) a Two-choice Reaction Time (TCRT) test where the subject had to choose, by tapping the screen with a stylus, whether a previously, briefly displayed numeral on the screen was either a "2" or a "3" *or *a "4" or a "5"; and (c) a Matching-to-Sample (MTS) test where the subject had to indicate with the stylus which of two specific patterns of squares had previously been displayed on the screen. The KSS was used to probe self-assessments of sleepiness [24]. Subjects performed tests every 15 minutes (three times for every light-and-dark condition) throughout a four-hour session. Tests were performed while subjects were sitting comfortably in a chair, either adjacent to the light box or in the dark anteroom.

PVT and KSS data were downloaded to an Excel spreadsheet for subsequent analyses using the BCDataMan 2.0.10 software from Behavioral Neuroscience Systems LLC. Both a response time and a score were determined for the TCRT and MTS tasks. Both scores were calculated by dividing the percentage of correct responses by the mean response times for each test during a given recording period.

### Melatonin

Melatonin, a hormone produced at night and under conditions of darkness, is used as a marker of circadian system timing. In darkness, melatonin levels rise in the evening, reach a peak between 02:00 and 04:00 and decline to daytime levels between 07:00 and 09:00. Light can attenuate the rate of melatonin production during the nighttime; depending upon the characteristics of light exposure (intensity, spectrum, duration), melatonin synthesis will continue to rise, but at a slower rate, in the early part of the night and continue to fall, but at a faster rate, toward the end of the night. In the current study, 1 to 2 ml saliva samples were collected at the end of each dark and each light exposure period using the Salivette system from Alpco Diagnostics (four samples per session per subject). The vessels containing the suspended saliva-impregnated cotton swabs were then spun in a centrifuge at 1000 × g for five minutes, causing the saliva to collect at the bottom of the centrifuge vessel. Saliva samples were frozen for transport to a laboratory for melatonin radioimmunoassay (Pharmasan, Osceola, WI). The sensitivity of the saliva assay was 0.7 pg/ml and the intra- and inter-assay coefficients of variability (CVs) were 12.1% and 13.2%, respectively.

### EEG and ECG

The Biosemi ActiveTwo system with active electrodes was used for EEG recordings. This system is battery powered, minimizing electrical interference from alternating current (ac) during recording sessions. Electrodes were placed on subjects' scalps according to the International 10-20 system at Oz, Pz, Cz, and Fz [25]. Two additional electrodes serving as virtual reference electrodes for those attached to the scalp were attached to the right and to the left earlobes. Another electrode was placed approximately 5 cm below the left clavicle to measure an electrocardiogram (ECG) signal.

Near the end of each dark and each light exposure period, just before collecting saliva samples, the scalp electrodes on each subject were attached to the EEG recording system. Six minutes of data were collected: three one-minute periods with the subject's eyes closed alternating with three one-minute periods with the eyes open. When the eyes were open and subjects were not sitting at the light box (dark condition), the subjects were asked to fixate on a specific marked point approximately one meter away. Similarly, when sitting at the light box, subjects fixated on specific point on the far wall of the box approximately 0.6 m away. Subjects were visually monitored by an experimenter to ensure compliance with the protocol.

The EEG signals were sampled at 16384 Hz and then low-pass filtered and downsampled to 2048 Hz for electronic storage by the Biosemi system. All subsequent EEG data processing and analyses were performed with Matlab, version R2008a, by The Mathworks™. The signals recorded from the two reference channels were averaged and these values were subtracted from those obtained from all of the other channels. The direct current (dc) offset of each channel was eliminated by subtracting the mean value of each channel from itself. A low-pass finite impulse response (FIR) filter (f-_3dB _= 50 Hz) was applied and the data were downsampled to 512 Hz. Then a high-pass, 3^rd ^order Butterworth filter (f-_3dB _= 4 Hz) was applied to the downsampled signals from each channel to eliminate slow trending in the data.

Another program divided the filtered data into 5-second epochs, segregated by periods when the eyes were open and when they were closed during the six-minute recording period. Eye blink artifacts were eliminated by removing epochs from all channels where voltage fluctuations of any epoch exceeded ± 100 μV. A Blackman window followed by a fast Fourier transform (FFT) was then applied to the data segments. This process yielded spectral power distributions from 1-50 Hz. The power spectra for each one-minute segment were then combined to give an average spectral power distribution for each trial. The relative power levels for eyes open in the theta (5-7 Hz), alpha-theta (5-9 Hz), alpha (8-12 Hz), beta (12-30 Hz), and gamma (30-50 Hz) ranges were calculated as a percentage of overall power from 1-50 Hz [4]. These calculations were not performed for those intervals when the eyes were closed because these data were only used for the AAC (below).

The AAC was computed from the ratio of the alpha power of eyes closed to the alpha power of eyes open for each of the three sessions in the six-minute recording period [26]. The three AAC values for a single measurement period were then averaged to arrive at an AAC value for that condition. One AAC value was calculated for each of the four channels.

The ECG data were digitally processed the same way as the EEG data up to the high-pass filtering. For the ECG analysis, the high-pass filtering -3dB cut-off was lowered to 0.2 Hz. Heart rates corresponding to the filtered ECG data were determined by two methods: 1) by taking the FFT of the ECG, whereby the frequency having the peak power within the range from 40 to 120 beats/minute is the heart rate, and 2) determining the mean elapsed time between successive QRS complexes of the ECG. The QRS complex represents ventricular depolarization. It is called a complex because there are three different waves in it (Q-wave, R-wave, and S-wave). The QRS complexes were located by the first derivative of the ECG falling below a negative threshold value (corresponding to the R-wave to S-wave transition) after individual normalization of the first derivative of the ECG. The values of the first derivative of the ECG at the QRS complexes were typically five to 10 times greater in magnitude than at any other part of the waveform, which enabled this simple algorithm to reliably find the QRS signatures. To further guard against spurious values and artifacts in the ECG data from affecting the mean heart cycle period and corresponding heart rate, only those periods between the 5% and 95% quantiles were included in the calculations of the reported means.

## Results

### EEG

Since the *a priori *hypothesis was that the circadian system mediated light-induced nocturnal alertness, the experiment was designed so that the two levels of red light exposure would serve as controls for the two levels of blue light exposure. Initially then, a pair of repeated measures ANOVAs (two [light spectra] by two [light levels] by four [channels]) was performed, one using the percent power in the EEG recordings for the alpha frequency range (8-12 Hz) and the other using the percent power for the beta frequency range (12-30 Hz), as recorded from four scalp electrode channels (Oz, Pz, Cz and Fz). There was no significant main effect of light spectra (F_1,13 _= 0.005, ns, and F_1,13 _= 1.09, ns, for alpha and beta power, respectively) or of light levels (F_1,13 _= 0.03, ns, for both measures), nor was the interaction between these independent variables statistically significant for either dependent variable (F_1,13_= 3.27, ns, and F_1,13 _= 3.65, ns, for alpha and beta, respectively). There was a significant effect of channels (F_3,39 _= 38.5, p < 0.0001, for alpha and F_3,39 _= 4.33, p < 0.01, for beta). As a check against potentially large individual differences masking the underlying treatment effects of interest, a second pair of ANOVAs was performed comparing the difference, for each subject, in alpha power and the difference, for each subject, in beta power between measurements made in the dark and in the subsequent light conditions. Again, there were no significant main effects for light spectra (F_1,13 _= 0.16, ns, and F_1,13 _= 0.89, ns, for alpha and beta, respectively) or light levels (F_1,13 _= 0.61 and F_1,13 _= 0.19, ns, for alpha and beta, respectively) nor was the interaction statistically significant using the differences in EEG power for either the alpha or the beta frequencies (F_1,13 _= 2.78, ns, for both frequencies). Again, however, there was a significant effect of channels.

These findings show, in effect, that light of either spectrum or either level is equally effective for inducing nocturnal alertness because there was no statistically reliable difference between the blue light and the red light exposures at either light level. In other words, the EEG power averages in the alpha frequency range and in the beta frequency range were statistically the same for all four of the lighting conditions. Importantly, the relative alpha power averages obtained under each of the four lighting conditions were always lower than those obtained in the preceding dark conditions, and the relative beta power averages were always higher than those obtained in the previous dark conditions.

Since there was no difference among the four lighting conditions in their effectiveness for inducing alertness at night compared to the previous darkness, the aggregated effects of light versus dark were examined using a third pair of repeated measures ANOVAs (two [dark versus light] by two [first and second sessions within the same night] by two [first and second nights] by four [channels]) was performed using the relative alpha power and the relative beta power as dependent variables. There was a significant main effect of dark versus light for both the alpha and the beta frequencies (F_1,13 _= 6.34, p = 0.03 for alpha and F_1,13 _= 17.2, p = 0.001 for beta). No other term in the two ANOVAs interacted with this key variable. This analysis demonstrates, as inferred indirectly from the initial ANOVAs, that, simply, the four lighting conditions had an alerting effect on subjects.

**Figure 3:**
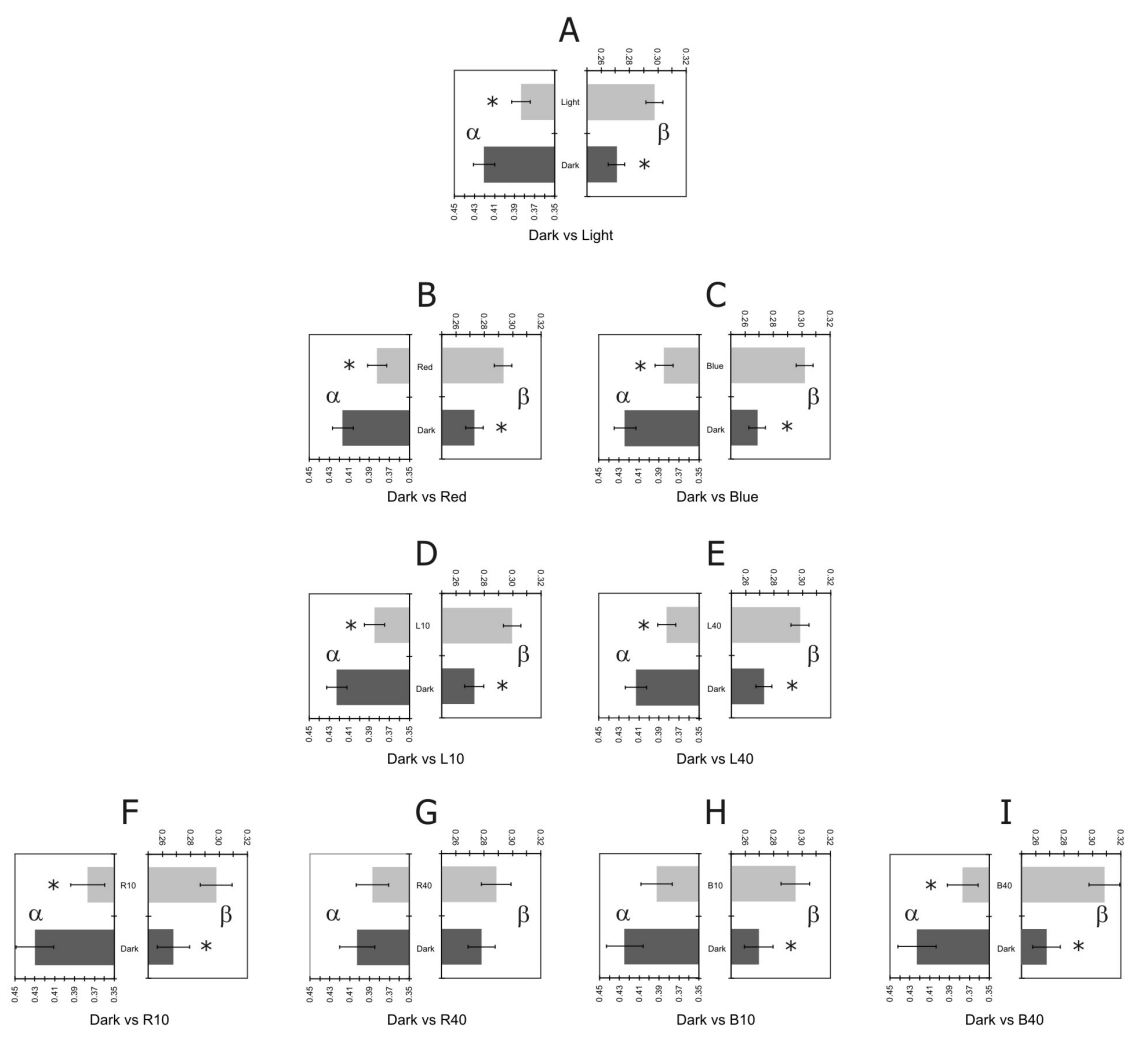
**Average relative (percent) power for alpha frequencies and beta frequencies for each lighting condition**. Higher levels of EEG alertness were associated with light than with dark; see text for details. Each pair of panels (A through I) represents the average relative (percent) power for alpha frequencies (α) and beta frequencies (β) obtained for a set of lighting conditions relative to those recorded during the corresponding set of preceding dark conditions. Error bars represent ± standard error of the mean (s.e.m.) for each condition. Asterisks represent significant differences (p < 0.00625). Panel A compares α and β obtained from all lighting conditions compared to α and β from the preceding dark conditions. Panels B and C compare α and β for the two light colors relative to α and β for the preceding dark conditions. Panels D and E compare α and β for the two light levels relative to α and β for the preceding dark conditions. Panels F through I compare α and β for the four combinations of light color and light level relative to α and β for the preceding dark conditions.

Since there were no significant interactions between the variable dark versus light and any of the other variables in the first two ANOVAs, the aggregate alpha power means for dark and for light and the aggregate beta power means for dark and for light were determined. These averages are shown in the top pair of panels in Figure 3; the comparison between light and dark for the alpha frequencies is shown in the left panel and for the beta frequencies in the right panel. To more precisely understand the effects of light spectra and light levels on nocturnal alertness, the aggregated mean alpha and beta power values for light and for dark were decomposed into smaller averages whereby, for example, the mean alpha power for red could be compared to the mean alpha power for the associated dark condition preceding that light condition. Figure 3 shows, in each pair of panels, how the aggregated mean values from all four channels for light and for dark were broken down. As this figure illustrates, eight pairwise comparisons were performed. To correct for multiple comparisons, the criterion alpha level (i.e., p < 0.05) was adjusted in accordance with the Bonferroni/Dunn method to p < 0.00625. Two-tail paired t-tests were performed for the alpha power and for the beta power using the combined data from all four channels.

The first t-test showed that the power in the alpha frequencies from all channels for combined dark conditions was higher than that for the combined light conditions (p < 0.0001); the power in the beta frequencies for the combined dark conditions was lower than the beta power for the combined light conditions (p < 0.0001). These two statistical comparisons simply mirror the results of the third ANOVA where the main effect of the variable dark versus light was significant. These effects are illustrated in the pair of panels labeled A in Figure 3; the comparison between light and dark for the alpha frequencies is shown in the left panel and for the beta frequencies in the right panel.

With regard to the combined red light conditions compared to the combined dark conditions preceding the red light exposures, the power in both alpha and beta frequencies were significantly different, in the expected direction (alpha, p < 0.0001; beta, p = 0.0002). These effects are illustrated in the pair of panels labeled B in Figure 3. The combined blue light conditions were significantly different than the combined dark conditions that preceded the blue light conditions for both alpha and beta power, in the expected direction (alpha, p < 0.0001; beta, p < 0.0001). These effects are illustrated in the pair of panels labeled C in Figure 3. Data for the 10 lx conditions were significantly different, in the expected direction, than the data associated with the preceding dark conditions using both alpha power (p < 0.0001) and beta power (p < 0.0001), as illustrated in the pair of panels labeled D in Figure 3. Similarly, the data for the 40 lx conditions were significantly different, in the expected direction, than the data associated with the preceding dark conditions for both alpha power (p = 0.0002) and beta power (p < 0.0001), as shown in the pair of panels labeled E in Figure 3.

Alpha power was higher for the dark condition preceding the blue-10 lx than for the blue-10 lx condition, although this difference (p = 0.007) did not reach the adjusted alpha criterion. The difference in alpha power was, however, significantly higher for the dark condition preceding the blue-40 lx condition than for the blue-40 lx condition (p < 0.0001). These differences are illustrated in the left panels of H and I, respectively, in Figure 3. For beta power, the same ordering effects were observed, but in the opposite, expected direction; beta power was significantly lower for the dark condition preceding the blue-10 lx than for the blue-10 lx condition (p = 0.0006), and lower for the dark condition preceding the blue-40 lx condition than for the blue-40 lx condition (p < 0.0001), as illustrated in the right panels of H and I, respectively, in Figure 3. Power in the alpha and beta frequencies were not significantly different for the red-40 lx condition and the previous dark condition (panel G of Figure 3), whereas power in both, alpha and beta frequencies were significantly different in the red-10 lx condition compared to the preceding dark condition (p < 0.0001 for alpha and beta) (panel F of Figure 3). The findings for the blue light conditions were expected, but those for the red light conditions were not expected; a greater effect should have been seen for the 40 lx exposure than for the 10 lx exposure. This lack of statistical significance may, in part at least, reflect the lower (p < 0.05) alpha power level associated with the dim condition preceding the R40 condition than with any of the other the dim condition.

A repeated measures ANOVA (two [dark versus light] by two [first and second sessions within the same night] by two [first and second nights] by four [channels]) was performed using the AAC values computed from the alpha power with the eyes closed to the alpha power with the eyes opened (data not shown). Several maximum amplitude criteria were applied to the raw alpha power (47 μV, 75 μV, 100 μV) to minimize artifacts in the data (however, AAC for one subject could not be determined, so the ANOVAs included data for 13 subjects). As expected, the channels (F_3,36 _= 21.28, p < 0.0001) were statistically different. The main effect of night (F_1,12 _= 5.61, p = 0.04) was also statistically significant. From this measure it would seem that subjects were more tired on the second night than on the first night. There was a statistically significant interaction between night and channel (F_3,36 _= 3.83, p = 0.02). This result reflects the fact that the differences in AAC among the four channels were smaller on the second night than on the first night. The interaction between light and channel (F_3,36 _= 3.21, p = 0.03) was also statistically significant. Parallel to the night by channel interaction, the differences in AAC among the four channels were smaller for the light condition than for the dark condition. These significant interactions do not have any obvious implication and since there was no main effect of light, the findings from the AAC will not be discussed further.

### ECG

As mentioned in the Methods section, the heart rate data were analyzed using two different techniques, FFT and QRS. Given the results of the series of statistical analyses conducted for the EEG data, two repeated measures ANOVAs (two [dark versus light] by two [first and second sessions within the same night] by two [first and second nights]) was performed using data from both the FFT and the QRS techniques. Only the main effect of sessions was statistically significant (F_1,13 _= 16.3, p = 0.004 using the FFT technique; F_1,13 _= 12.0, p = 0.005 using the QRS technique) indicating, as might be expected, that heart rate was significantly lower during the second session (later at night) than during the first. Although there was no statistically significant effect of dark versus light, paired one-tail t-tests were conducted to determine if specific lighting conditions were statistically different than their previous dark condition. The first t-test showed a statistically significant increase in heart rate after exposure to the blue-40 lx condition compared to the prior dark period for both techniques (FFT, p = 0.003; QRS, p = 0.04). The mean ± standard error of the mean (s.e.m.) heart rate in the blue-40 lx condition was 64.4 ± 1.2 for the FFT and 63.6 ± 1.1 for the QRS. The mean ± s.e.m. heart rate in the dark preceding the blue-40 lx condition was 61.8 ± 1.0 for the FFT and 62.3 ± 1.1 for the QRS. For the red-40 lx condition, only the QRS method showed a significant increase in heart rate relative to the previous dark condition (p = 0.04). The mean ± s.e.m. heart rate in the red-40 lx condition was 61.9 ± 1.1 for the QRS, and in the dark preceding the red-40 lx condition it was 61.1 ± 1.1. No significant differences in heart rate were found using either technique at the lower level (10 lx) of either blue or red light. Although the ECG results are weaker than those from the EEG data, they do suggest that sufficient irradiance at the cornea of either red or blue light exposure can increase alertness at night.

### PVT and KSS

Each of the outcome measures obtained from the PDA (RT, TCRT, MTS, and KSS) were submitted to the same series of repeated measures ANOVAs as the ECG variables, with one additional factor, trial: (i.e., two [dark versus light] by two [first and second sessions within the same night] by two [first and second nights] by three [trials]). Three successive trials were conducted during every light and every dark condition, one at the beginning, one in the middle, and one at the end of the 60-minute light exposure or dark condition. One subject did not follow instructions; that subject's data were not included in the analyses.

For KSS, the main effects of session (F_1,12 _= 46.14, p < 0.0001), trial (F_2,24 _= 22.06, p < 0.0001), and light (F_1,12 _= 34.98, p < 0.0001) were statistically significant, as was the session by light interaction (F_1,12 _= 11.19, p = 0.006). The three main effects demonstrate increasing sleepiness throughout the experiment. The second session was associated with higher values of KSS than in the first session; Paired two-tail t-tests showed that KSS values associated with the second trial and with the third trial were significantly higher than those in the first trial (p < 0.0004 and p < 0.0001, respectively) although values for the second trial were not significantly different than those associated with the third trial. KSS values were significantly higher (p < 0.0001) in the light exposure condition than in the dark, but since every light exposure condition followed a dark condition, even the significant main effect of light very likely reflects growing sleepiness by the subjects throughout the experiment. Interestingly, however, the significant interaction between session and light suggests that light, in fact, served as a countermeasure for fatigue: a two-tail paired t-test revealed that the difference in reported sleepiness between session 1 and session 2 was significantly greater (p < 0.0001) in the dark than in the light.

In terms of the performance measures, only the TCRT task showed a significant main effect for trial with response time (F_2,24 _= 6.06, p < 0.007) and with score (F_2,24 _= 8.43, p < 0.002). For both TCRT measures, performance was consistently worse as the trials progressed (i.e., response time increased and score decreased). Consistent with the KSS data, these results indicate that fatigue played an important role in this study. Moreover, the trial by night interaction was statistically significant for both TCRT measures (F_2,24 _= 4.98, p < 0.02, for response time; F_2,24 _= 4.08, p < 0.03, for score), suggesting that, consistent with the AAC results, fatigue played a greater role in the second night than in the first night; paired two-tail t-tests showed that the difference in TCRT for response time from the first trial to the third trial was significantly greater (p = 0.02) on second night than on the first night, but the difference in TCRT for score did not reach significance (p = 0.1). No effects of light exposure, either among the different light exposure conditions or relative to the previous dark conditions, were demonstrated by any of the performance measures.

### Melatonin

Melatonin suppression for each lighting condition was calculated for every subject using the following formula: 1 - (melatonin in the light/melatonin in the dark). If light exposure is a strong stimulus to the circadian system, it will suppress melatonin levels below those measured in the previous dark condition, whereas if it is a weak stimulus, light exposure may not fully counteract the natural rise in melatonin levels that occurs during the early nighttime, in which case negative suppression will be observed. The suppression values associated with three of the four lighting conditions were then determined from those obtained from all 14 subjects; melatonin levels could not be detected from one subject's saliva sample for the blue-40 lx condition, so the average suppression for this condition was based on 13 subjects. Using these data, a two [light spectra] by two [light levels] repeated measures ANOVA was performed; neither the main effects were significant (light color, F_1,12 _= 0.3, ns; light level F_1,12 _= 1.9, ns), nor was the interaction (F_1,12 _= 1.1, ns). However, melatonin concentrations were suppressed by 18% ± 15% (average ± s.e.m.) after exposure to the blue-40 lx condition relative to the preceding dark condition, whereas negative suppression values were determined for the other three lighting conditions (-62% ± 33% for red-10 lx, -39% ± 24% for red-40 lx, and -96% ± 82% for blue-10 lx). In other words, melatonin levels were significantly lower for the blue-40 lx condition than for the dark conditions preceding the blue-40 lx exposures, supporting the literature [12] that this lighting condition was the strongest circadian stimulus. In fact, post hoc one-tail paired t-tests revealed a significantly higher level of melatonin suppression for the blue-40 lx condition than for either the red-40 lx condition (p = 0.03) or the red-10 lx condition (p = 0.02). There was no significant difference between the blue-10 lx condition and the blue-40 lx condition (p = 0.09).

It must be noted that, because of the counterbalanced experimental design, the average suppression values used in these statistical analyses were determined from combined suppression values obtained at two different circadian times. In the counterbalanced experimental design, half the subjects would have been presented with the 10 lx conditions before the 40 lx conditions, and half the subjects would have been presented with the blue light conditions before the red light conditions. Thus, average suppression values for every lighting condition were based upon data obtained during both the early and the later exposure times in the sessions. Since the rate of melatonin synthesis changes throughout the night, melatonin suppression values will be differentially affected by the sample time. Specifically, the same light stimulus presented early in the night will result in less suppression than when it is presented later in the night because the rate of melatonin synthesis is high in the early night, reaches a peak value in the middle of the night, and then decreases until the end of the night [27].

## Discussion

Nocturnal alertness as measured by ECG and EEG is affected by light, but the results presented here suggest that a pathway other than the circadian system may affect light-induced nocturnal alertness because red light as well as blue light was effective. Heart rate measured with both the FFT and the QRS techniques increased significantly relative to the dark conditions for the higher level (40 lx) of both blue and red light, although the lower level (10 lx) of the two spectra was not effective. In terms of the EEG data, both red and blue light (combining both 10 lx and 40 lx) reduced alpha power and increased beta power levels relative to their respective preceding dark conditions. Similarly, both 10 lx and 40 lx of light (combining both blue and red) had a significant impact on alpha and on beta relative to their respective preceding dark conditions. Although both 10 lx and 40 lx were effective for the blue light, only 10 lx, but not 40 lx of red light significantly affected the EEG recordings compared to their preceding dark conditions. This apparent dose intransitivity for the red light conditions remains unexplained and is somewhat implausible, although it is conceivable that there is an optimum irradiance of red light for alertness (i.e., red-10 lx), but this hypothesis seems rather unlikely and these results definitely demand further study.

Although there was some evidence that light mitigated the growing sleepiness in the experiment (i.e., the statistically significant session by light interaction), both the KSS and the PVT results did not support the inference that light is a robust countermeasure for subjective sleepiness or for performance decrement, thus suggesting that for this protocol electrophysiological measures of alertness can be more sensitive than behavioral measures of alertness.

## Conclusion

Based mainly on the EEG and ECG data, the present results are, to a limited extent, consistent with previous findings showing that light of sufficient corneal irradiance increases alertness at night [1-9]. There is previous compelling evidence that light-induced stimulation of the circadian system increases alertness at night, but the present results suggest that this effect is not mediated only by the circadian system, implicating other mechanisms through which light can also increase nocturnal alertness. It is important then to determine if these inferred mechanisms are independent of the circadian system or interact with it by conducting light studies at different circadian times.

## Authors' contributions

MGF participated in the conception and the design of the experiment, data collection, data analyses and interpretation, and manuscript writing. AB participated in the data analyses and interpretation and reviewed the manuscript. BP participated in data collection, analyses, and drafted sections of the manuscript. MR participated in the conception and design of the experiment, data interpretation and manuscript writing. All authors read and approved the final manuscript.

## References

[B1] Badia P, Myers B, Moecker M, Culpepper J (1991). Bright light effects on body temperature, alertness, EEG and behavior. Physiol Behav.

[B2] Boyce P, Beckstead J, Eklund N, Strobel R, Rea M (1997). Lighting the graveyard shift: The influence of a daylight-simulating skylight on the task performance and mood of nightshift workers. Light Res Technol.

[B3] Cajochen C, Krauchi K, Danilenko KV, Wirz-Justice A (1998). Evening administration of melatonin and bright light: interactions on the EEG during sleep and wakefulness. J Sleep Res.

[B4] Cajochen C, Zeitzer J, Czeisler C, Dijk D (2000). Dose-response relationship for light intensity and ocular and electroencephalographic correlates of human alertness. Behav Brain Res.

[B5] Campbell SS, Dawson D (1990). Enhancement of nighttime alertness and performance with bright ambient light. Physiol Behav.

[B6] Daurat A, Foret J, Benoit O, Mauco G (2000). Bright light during nighttime: effects on the circadian regulation of alertness and performance. Biol Signals Recept.

[B7] Eastman CI, Liu L, Fogg LF (1995). Circadian rhythm adaptation to simulated night shift work: effect of nocturnal bright-light duration. Sleep.

[B8] Lowden A, Akerstedt T, Wibom R (2004). Suppression of sleepiness and melatonin by bright light exposure during breaks in night work. J Sleep Res.

[B9] Figueiro M, Rea M, Boyce P, White R, Kolberg K (2001). The effects of bright light on day and night shift nurses' performance and well-being in the NICU. Neonatal Intens Care.

[B10] Brainard G, Hanifin J, Greeson J, Byrne B, Glickman G, Gerner E, Rollag M (2001). Action spectrum for melatonin regulation in humans: Evidence for a novel circadian photoreceptor. J Neurosci.

[B11] Thapan K, Arendt J, Skene DJ (2001). An action spectrum for melatonin suppression: evidence for a novel non-rod, non-cone photoreceptor system in humans. J Physiol.

[B12] Rea M, Figueiro M, Bullough J, Bierman A (2005). A model of phototransduction by the human circadian system. Brain Res Rev.

[B13] Cajochen C, Munch M, Kobialka S, Krauchi K, Steiner R, Oelhafen P, Orgul S, Wirz-Justice A (2005). High sensitivity of human melatonin, alertness, thermoregulation and heart rate to short wavelength light. J Clin Endo Met.

[B14] Figueiro MG, Bullough JD, Bierman A, Fay CR, Rea MS (2007). On light as an alerting stimulus at night. Acta Neurobiol Exp (Wars).

[B15] Saper CB, Cano G, Scammell TE (2005). Homeostatic, circadian, and emotional regulation of sleep. J Comp Neurol.

[B16] Saper CB, Lu J, Chou TC, Gooley J (2005). The hypothalamic integrator for circadian rhythms. Trends Neurosci.

[B17] Saper CB, Scammell TE, Lu J (2005). Hypothalamic regulation of sleep and circadian rhythms. Nature.

[B18] Stone NJ (2003). Environmental view and color for a simulated telemarketing task. J Environ Psychol.

[B19] Hill RA, Barton RA (2005). Psychology: red enhances human performance in contests. Nature.

[B20] Elliot AJ, Maier MA, Moller AC, Friedman R, Meinhardt J (2007). Color and psychological functioning: the effect of red on performance attainment. J Exp Psychol Gen.

[B21] Roenneberg T, Wirz-Justice A, Merrow M (2003). Life between clocks: daily temporal patterns of human chronotypes. J Biol Rhythms.

[B22] Thorne DR, Johnson DE, Redmond DP, Sing HC, Belenky G, Shapiro JM (2005). The Walter Reed palm-held psychomotor vigilance test. Behav Res Methods.

[B23] Lamond N, Dawson D, Roach GD (2005). Fatigue assessment in the field: validation of a hand-held electronic psychomotor vigilance task. Aviat Space Environ Med.

[B24] Akerstedt T, Gillberg M (1990). Subjective and objective sleepiness in the active individual. Int J Neurosci.

[B25] American Electroencephalographic Society (1991). Guidelines for standard electrode position nomenclature. J Clin Neurophysiol.

[B26] Alloway CE, Ogilvie RD, Shapiro CM (1997). The alpha attenuation test: assessing excessive daytime sleepiness in narcolepsy-cataplexy. Sleep.

[B27] Meltzer JA, Zaveri HP, Goncharova II, Distasio MM, Papademetris X, Spencer SS, Spencer DD, Constable RT (2008). Effects of working memory load on oscillatory power in human intracranial EEG. Cereb Cortex.

